# Deterministic clustering of the available chemical space

**DOI:** 10.1186/1758-2946-5-S1-P53

**Published:** 2013-03-22

**Authors:** Philipp Thiel, Lisa Peltason, Christian Ottmann, Oliver Kohlbacher

**Affiliations:** 1 Chemical Genomics Centre of the Max Planck Society, Dortmund, 44227, Germany; 2 F. Hoffmann-La Roche AG, CH-4070 Basel, Switzerland; 3 Applied Bioinformatics, Center for Bioinformatics, Quantitative Biology Center and Dept. of Computer Science, University of Tübingen, Tübingen, 72076, Germany

## 

Clustering of compound libraries using 2D binary fingerprints is a fundamental task in chemoinformatics and various methods have been described to solve it [1]. These methods can roughly be grouped into deterministic and non-deterministic approaches with two key-characteristics distinguishing them. First, the algorithmic complexity of deterministic approaches is more demanding whereas the non-deterministic methods often try to overcome this drawback by using heuristics to save time and memory. Second, deterministic clustering algorithms, especially agglomerative hierarchical techniques have been shown to yield good results and often perform better than non-deterministic approaches [2]. As a consequence, clustering of small to medium sized libraries with up to 1 million compounds is regularly performed using deterministic techniques whereas libraries comprising millions of compounds are mostly clustered using heuristics like k-means [3].

Here, we present a deterministic approach for clustering huge compound libraries based on all pairwise compound similarities. For this purpose, we use an extremely fast and flexible algorithm for similarity calculations, which we have developed to be purely CPU-based thus having no need for any specialized hardware. Using this similarity method, we implemented a workflow with the following steps. First, we create a set of unique input fingerprints by filtering duplicates that are then stored and finally remapped onto their representative clusters. Second, we calculate all pairwise similarities to construct a similarity network by applying a fixed Tanimoto threshold to select the edges to be inserted into the network. From this similarity network the connected subgraphs are extracted and forwarded to the last step. Finally, connected subgraphs exceeding a predefined size are hierarchically clustered.

As a result, we show that our algorithm for similarity calculation is competitive to recently published CPU-based methods and can perform up to 380 million Tanimoto calculations per second on a current desktop computer. This efficient method allows our workflow to process medium to large libraries on current desktop computers within minutes. To finally demonstrate the power of our clustering workflow, we processed the commercially available chemical space comprising about 17 million compounds [4]. The entire clustering workflow took 63 hours on a compute server using 64 cores and 100 GB main memory to complete.
